# Psychometric Properties of a Spanish Version of the Basic Needs Satisfaction in Sports Scale

**DOI:** 10.3389/fpsyg.2019.02816

**Published:** 2019-12-13

**Authors:** H. Antonio Pineda-Espejel, Icela López Gaspar, Andrea Carmen Guimaraes, Sonia Martínez Zavala, Raquel Morquecho-Sánchez, Verónica Morales-Sánchez, Estelio Henrique Dantas

**Affiliations:** ^1^Facultad de Deportes, Universidad Autónoma de Baja California, Mexicali, Mexico; ^2^Facultad de Idiomas, Universidad Autónoma de Baja California, Mexicali, Mexico; ^3^Departamento das Ciências da Educação Física e Saúde, Universidade Federal de São João del-Rei, São João del Rei, Brazil; ^4^Facultad de Organización Deportiva, Universidad Autónoma de Nuevo León, San Nicolás de los Garza, Mexico; ^5^Facultad de Psicología, Universidad de Málaga, Málaga, Spain; ^6^Federal University of Rio de Janeiro, Rio de Janeiro, Brazil

**Keywords:** test adaptation, validity, composite reliability, self-determination, sports

## Abstract

The Basic Needs Satisfaction in Sport Scale (BNSSS) is an instrument designed to measure the level of satisfaction of the three basic psychological needs (BPN) in sports, according to The Self-Determination Theory (SDT). The purpose of this research was to adapt BNSSS to Mexican Spanish and analyze its psychometric properties (factorial validity, factorial invariance, internal consistency, convergent validity, and nomological validity). Thus, 542 athletes (average age: 12.06 years; *SD* = 1.83) were asked to answer a set of questionnaires. Confirmatory factor analyses (CFA) supported both the structure of five related factors and the trifactorial structure after eliminating an item. Nevertheless, the reliability analysis indicated strong internal consistency, and the average variance extracted (AVE) from the subscales was acceptable except for the volition factor, thus supporting the trifactorial model. Scores derived from the instrument’s three-factors offered evidence of the criterion validity, through a positive and meaningful relation with enjoyment and subjective vitality. Moreover, results of multi-sample analysis supported that factorial structure is invariant between men and women. In conclusion, this BNSSS Spanish version displayed adequate psychometric properties, showing that it can be used to measure the three basic psychological needs.

## Introduction

The Self-Determination Theory (SDT; Deci and Ryan, 1985; Ryan and Deci, 2002) is a meta-theory of motivation, emotion, and human personality, which conceives humans as active, growth-oriented organisms. This theory has been used to explain and predict how motivation works in a variety of life contexts, including sports (Balaguer et al., 2008; Álvarez et al., 2009; Quested et al., 2013; Cantú-Berrueto et al., 2016), and physical activity (Rodrigues et al., 2018). This theory is the most influential theory in competitive sport motivation (Clancy et al., 2016), given that motivation can help explain aspects as the intention to continue training (e.g., Monteiro et al., 2018).

One of the main mini-theories of the SDT is the theory of basic psychological needs (BPN; Ryan and Deci, 2000, 2017), which provides the basis for describing the environmental characteristics that support or hinder the attempt of trying to control new situations, or that facilitate self-determination and well-being. It proposes that people have three inherent BPN. First, relatedness refers to the need to experience mutual care, acceptance, and concern from the people close to them; in other words, feeling connected or in unity with others. Second, competence is a sense of confidence and effectiveness in producing desired outcomes. Third, autonomy denotes experiencing high flexibility and low levels of pressure during one’s actions, and the feeling that it was performed voluntarily; in other words, perceiving that oneself is the origin or the source of behavioral self-regulation (Deci and Ryan, 1985).

Regarding the need for autonomy, Reeve et al. (2003) stated that this is a concept that links three qualities of experience. The most common quality is the *internal perceived locus of causality* (IPLOC) (deCharms, 1968; Deci and Ryan, 1985), which refers to a person’s beliefs that their actions are initiated and regulated by a personal force, or that the person is the origin of the behavior (deCharms, 1968). A second quality of autonomy is *volition*, which refers to being involved or not in an activity without pressures, meaning by one’s will (Deci et al., 1996). The third quality is the *perceived choice* that originates from a perception of making the decision flexibly (Reeve et al., 2003). These three qualities are important in sports context, for example, in one hand, athletes who highly appreciate their coach’s experience can choose to let the coach make the strategic decisions and still feel autonomous in the process. In the other hand, coaches could let the participants set their own personal goals, to encourage the psychological well-being (Reinboth and Duda, 2006).

According to the SDT, the BPN compose the necessary nutrients for the vital functioning of the organism; so that the three needs are essential for growth and development (Ryan and Deci, 2004). It is expected that a great satisfaction of psychological needs tends to produce great internalization of behavior and high levels of self-determined motivation, as well as to improve well-being (Ryan and Deci, 2002). This thought has been supported in the sports context by some studies showing that the satisfaction of the three psychological needs predict autonomous motivation (Álvarez et al., 2009; González et al., 2015; Cantú-Berrueto et al., 2016; Heuzé et al., 2018), enjoyment (Quested et al., 2013; Monteiro et al., 2018) and subjective vitality (González et al., 2015; Garza-Adame et al., 2017), being the latter regarded as a general organismic concept of eudaimonic well-being (Ryan and Deci, 2004). Subjective vitality is considered a positive feeling of having available energy emanate from the self (Ryan and Frederick, 1997), and together with enjoyment in sports practice are critical to physical and psychological functioning.

Researchers adapted some instruments from other fields to the sport context. In the other hand, because of the lack of sport-specific instruments, some Spanish instruments designed for physical activities purposes have been use for sports researches. For example, the following instruments have been used: the Basic Psychological Needs in Exercise Scale (Moreno et al., 2008), the Perceived Autonomy in Sport Scale (Balaguer et al., 2008), the Need for Relatedness Scale (Balaguer et al., 2008), or the Psychological Need Satisfaction in Exercise Scale (Moreno-Murcia et al., 2011). The adaptation from physical activity to sports context can be problematic because the satisfaction of basic needs can be characterized differently (Ng et al., 2011).

In order to have a better understanding of either the background and the consequences of psychological needs satisfaction in the sport context, specific instruments of this context are necessary. The Basic Needs Satisfaction in Sport Scale (BNSSS) is an instrument developed in the context of sports that includes the measurement of the three psychological needs besides integrating two qualities of the experience of autonomy (i.e., perceived choice and IPLOC) which were not examined in some previous instruments (Ng et al., 2011). The BNSSS demonstrated an adequate validity of a five-factor structure, supporting the three qualities of autonomy separate measure. This instrument suggest that volition and IPLOC items do not discriminate adequately. According to the five-factors, these have presented good reliability (e.g., Mahoney et al., 2014). This instrument has been adapted and validated both in the Spanish (De Francisco et al., 2018) and Portuguese (Andrade et al., 2018) versions. The Spanish version, the five-factors structure (competence satisfaction and relatedness satisfaction, volition, perceived choice, and IPLOC) were tested only with team sports. The analyses showed that the items of IPLOC and volition do not differentiated between them. The Portuguese version tested a three-factor structure (competence satisfaction, relatedness satisfaction, and autonomy satisfaction), and did not include all the original items.

Within the SDT, it is hypothesized that the three BPN are universal through gender, cultures and ages. So, one of the theoretical objectives would be its use for all ages, genders and cultures (Ryan and Deci, 2002). In addition, there is no evidence for discriminant validity between two-factors (IPLOC and volition) in the BNSSS Spanish version (De Francisco et al., 2018). Therefore, the objective of this work is to adapt the BNSSS (Ng et al., 2011) to Spanish spoken in Mexico and to analyze its psychometric properties (factorial validity, factorial invariance, internal consistency, convergent validity, and nomological validity) with a sample of child–adolescent athletes.

Supported by the contributions of Reeve et al. (2003) and Ng et al. (2011), a first model of five-factors is hypothesized: competence satisfaction, relatedness satisfaction, volition, perceived choice, and IPLOC; the last three are first-order factors that are theorized as qualities of autonomy. Then, according to the BPN, a three-factor model is considered: competence satisfaction, relatedness satisfaction, and autonomy satisfaction. The autonomy satisfaction is a latent construct integrated by the three observed qualities (volition, perceived choice, and IPLOC).

## Materials and Methods

### Participants

Through an intentional non-probabilistic sampling, 542 Mexican athletes of both genders participated (324 men and 214 women; four athletes did not indicate their gender); their age ranged from 8 to 15 years old (*M* = 12.06 years; *SD* = 1.83). They practiced different sports specialties (e.g., athletics, basketball, soccer, gymnastics, swimming) and were members of their respective national federations. The reported a sporting experience of 3 years (*SD* = 2.57) and they trained for an average 120 min per week (*SD* = 0.76).

### Instruments

The satisfaction of BPN was measured with the BNSSS (Ng et al., 2011). It consists of 20 items, five items measure the perception of competence (e.g., “I am skilled at sport”), five items measure the perception of relatedness (e.g., “In sport, I have a close relationship with other people”), and 10 items measure the perception of autonomy. This latter is measured from three categories called volition (e.g., “I feel I participate in my sport willingly”), choice (e.g., “In sport, I get opportunities to make choices.”), and IPLOC (e.g., “In sport, I feel I am pursuing goals that are my own”). The answers are collected on a Likert scale ranging from *not at all true* (1) to *very true* (7). A higher score is interpreted as a greater satisfaction of the needs that the scale measures.

Subjective vitality was assessed using a Subjective Vitality Scale Spanish version (Castillo et al., 2017). It is composed of seven items that globally measure liveliness and energy subjective feelings. Athletes were asked to indicate the extent to which, in general, a series of statements are true for them (e.g., “I have energy and encouragement”). The answers were collected on a seven-point Likert scale ranging from *not at all true* (1) to *very true* (7).

To measure the enjoyment in sports practice, the Physical Activity Enjoyment Scale (PACES) Spanish version was used (Moreno et al., 2008). This instrument was adapted to sports context by modifying its stem from the original “When I am physically active.” to “When I’m training my sport.” followed by the 16 items that evaluate enjoyment in a direct manner (e.g., “I enjoy it”) and in reverse (e.g., “I feel bored”). The answers are collected on a Likert scale that ranges from *strongly disagree* (1) to *strongly agree* (5).

In addition, a section with questions about sociodemographic factors in terms of gender, age, training record (practiced sport, years of training, sessions per week, and daily training session length) was added.

### Process

In order to translate and adapt BNSSS to the Spanish spoken in Mexico, the inverse translation strategy, and the guidelines for the translation and adaptation of tests from one culture to another were used (Muñiz et al., 2013). For this process, the scale in English was translated into Spanish spoken in Mexico independently by two translators with high knowledge of both English and Spanish, and who consider the linguistic and cultural uniqueness of the Mexican context. The items were translated considering the equivalent concept of each phrase, not word into word. Afterward, the discrepancies of the translations were discussed by two sport psychology experts, making corrections in certain items until developing a first version of the instrument in Spanish spoken in Mexico.

Subsequently, this version was translated into English by a translator unrelated from the research group, emphasizing not only linguistic equivalence, but the conceptual and cultural equivalence as well. After that, the two versions, original and translation, were compared.

To analyze content validity, two psychology experts and a sports expert with experience validating instruments background reviewed the resulting version to ensure that the items were relevant, and that the writing was appropriate for the target population. Before obtaining the final version of the instrument, the scale was applied to a small group (*n* = 20) of children and teenagers athletes to verify the correct understanding of the items. They expressed to understand all the statements. Based on the comments of the people surveyed, the items were designed trying to maintain the semantic sense and the original structure.

The ethical approval of this work was provided by the Local Committee for Research and Ethics in Health Research # 204. The research was conducted following the ethical guidelines proposed by the American Psychological Association (APA). The first personal contact was with the coaches, to request authorization so that the athletes could participate in the study. The second contact was meeting with the parents or guardians of the athletes to inform them about the study and obtain their written authorization. The final application of questionnaires was carried out in the presence of the first author, who would solve any upcoming doubts and was careful not to skew possible answers. The athletes were reiterated of the anonymity, confidentiality, and sincerity of their answers. The approximate time to complete the questionnaires was 20 min.

### Data Analyses

A preliminary analysis was performed to detect outliers and calculate descriptive and univariate normal statistics (skewness and kurtosis). For factorial validity, confirmatory factor analyses (CFA) were made with the LISREL program 8.80. Although univariate normality does not guarantee multivariate normality, if all variables meet this requirement, then any departures from multivariate normality are usually inconsequential (Hair et al., 2018). For that reason, the maximum likelihood (MLE) was used as the estimation technique. This method has proven fairly robust to violations of the normality assumption. This offers a robust statistic of χ^2^ called Satorra–Bentler (S–Bχ^2^). The input matrices were co-variances and asymptotic co-variances.

The fit indexes absolute, incremental and parsimony were used to evaluate the model. Since the χ^2^ is sensitive to the size of the sample (Hu and Bentler, 1995), the adjustment of the model was evaluated with the addition of the Root Mean Square Error of Approximation (RMSEA) plus its 90% confidence interval. Also Non-Normative Fit Index (NNFI), Comparative Fit Index (CFI), and Parsimony Goodness-of-Fit Index (PGFI) were used. For RMSEA, lower or equal values at 0.05 and 0.08 were considered to show good and acceptable fit, respectively (Hair et al., 2018); while values that exceed 0.10 were considered undesirable (Browne and Cudeck, 1993). NNFI and CFI values equal to or greater than 0.90 indicated good fit (Hu and Bentler, 1999). The cut-off point of PGFI is 0.60 (Hair et al., 2018). Items that presented a factor loading lower than 0.50 were considered for elimination (Hair et al., 2018).

Analysis of metric equivalence through CFA multisample was applied to evaluate factorial invariance through gender. First, the CFA of each group was tested (men and women). Then, a base model was tested to analyze structural invariance. Afterward, even more restricted models were specified to examine measurement equality (invariance of factor loadings and intercepts across groups). To begin the analysis, covariance matrices, asymptotic covariance and means vector were used.

To assess differences between the adjustment of alternative models, it was suggested that differences equal to or lower than 0.01 between values of NNFI (ΔNNFI; Widaman, 1985) and CFI (ΔCFI; Cheung and Rensvold, 2002), indicate irrelevant practical differences. Also, differences in RMSEA values smaller than 0.015 between models indicate irrelevant differences (Chen, 2007).

In addition, convergent validity was obtained through composite reliability analysis (McDonald’s omega), where values higher than 0.70 showed good reliability (Hair et al., 2018), and the average variance extracted (AVE), where values higher than 0.50 indicates good fit (Hair et al., 2018). Lastly, correlation analyses were made to explore nomological validity, considering the dimensions of BNSSS as independent variables, and subjective vitality and enjoyment were considered as dependent variables. This analysis was performed using SPSS 22.0 program.

## Results

### Confirmatory Factor Analysis (CFA)

The first model (Model 1) tested the five-factor structure hypothesized from BNSSS: relatedness satisfaction, competence satisfaction, volition, perceived choice, and IPLOC. The last three are first-order factors, theorized as qualities in the experience of autonomy. The results of this model showed good fit to the data (Table 1). The items factor loading were significant (Table 2); however, an item from volition (“In sport, I feel that I am being forced to do things that I don’t want to do”) was significant (*p* < 0.05), but still below the criterion established (λ < 0.50). For this reason, this item was eliminated in an alternative model tested (Model 1A). When comparing incremental fit indexes between both models, the differences were trivial (see Table 1); as a consequence, the model including all items could be a better option. For Model 1, correlations among latent factors were between 0.17 and 0.89, considering that the discriminant validity between volition factors and IPLOC cannot be supported (see Table 3).

**TABLE 1:** Goodness of fit indexes of the models put to the test for the Basic Needs Satisfaction in Sport Scale (BNSSS).

	**SB χ ^2^**	***df***	**RMSEA (CI 90%)**	**NNFI**	**CFI**	**PGFI**	**Compared models**	**ΔRMSEA**	**ΔNNFI**	**ΔCFI**
Model 1	259.95^∗∗^	160	0.05 (0.04–0.06)	0.98	0.98	0.60				
Model 1A	201.52^∗∗^	142	0.04 (0.03–0.05)	0.98	0.99	0.60	Model 1 vs. Model 1A	0.01	<0.01	<0.01
Model 2	501.76^∗∗^	167	0.10 (0.09–0.11)	0.93	0.94	0.60				
Model 2A	392.17^∗∗^	149	0.08 (0.07–0.10)	0.95	0.95	0.61	Model 2 vs. Model 2A	0.02	>0.01	>0.01

*^∗∗^*p* < 0.001; *df* = degrees of freedom; CI = confidence interval; Model 1 (five-factor model); Model 1A (five-factor model without an item); Model 2 (higher order three-factor model); Model 2A (three-factor model without an item).*

**TABLE 2:** Weighting factors and descriptive statistics of the items of the Basic Needs Satisfaction in Sport Scale (BNSSS).

**Item**	***M***	***SD***	**Skewness**	**Kurtosis**	**λ**	**δ**
***Competence satisfaction***						
2. I can overcome challenges in my sport. (Puedo superar retos en mi deporte)	5.88	1.52	–1.47	1.62	0.76	0.42
5. I am skilled at my sport. (Soy hábil en mi deporte)	5.46	1.50	–0.82	0.06	0.91	0.17
9. I feel I am good at my sport. (Pienso que soy bueno/a en mi deporte)	5.65	1.51	–1.30	0.31	0.94	0.12
15. I get opportunities to feel that I am good at my sport. (Hay situaciones que me hacen sentir que soy bueno en mi deporte)	5.5	1.58	–1.11	0.85	0.79	0.38
18. I have the ability to perform well in my sport. (Tengo la habilidad para obtener buenos resultados en mi deporte)	5.68	1.44	–1.10	0.84	80	0.37
***Perceived choice***						
1. In my sport, I get opportunities to make choices. (En mi deporte, tengo oportunidades para elegir libremente)	5.16	1.76	–0.78	–0.23	0.61	0.63
7. In my sport, I have a say in how things are done. (En mi deporte, puedo dar mi opinión de cómo hacer las cosas)	4.35	2.07	–0.23	–1.16	0.75	0.43
13. In my sport, I can take part in the decision-making process. (En mi deporte, puedo participar en la toma de decisiones)	4.55	1.98	–0.35	–1.00	0.88	0.2.3
19. In my sport, I get opportunities to make decisions. (En mi deporte, tengo oportunidades para decidir)	4.82	1.81	–0.57	–0.47	0.86	0.26
***IPLOC***						
4. In my sport, I feel I am pursuing goals that are my own. (En mi deporte, siento que estoy persiguiendo mis propias metas)	5.89	1.42	–1.33	1.31	0.80	0.36
10. In my sport, I really have a sense of wanting to be there. (En mi deporte, realmente tengo la sensación de querer estar allí)	5.87	1.46	–1.21	0.65	0.86	0.26
12. In my sport, I feel I am doing what I want to be doing. (En mi deporte, siento que estoy haciendo lo que quiero hacer)	5.84	1.55	–1.24	0.58	0.87	0.24
***Volition***						
3. I feel I participate in my sport willingly. (Siento que participo en mi deporte de buena gana)	5.74	1.70	–1.34	0.85	0.67	0.55
8. In my sport, I feel that I am being forced to do things that I don’t want to do. (En mi deporte, siento que estoy siendo obligado/a a hacer cosas que no quiero hacer)	5.00	2.27	–0.66	–1.11	0.18^∗^	0.97
16. I choose to participate in my sport according to my own free will. (Siento que participo en mi deporte por voluntad propia)	5.81	1.63	–1.32	0.81	0.63	0.61
***Relatedness satisfaction***						
6. In my sport, I feel close to other people. (En mi deporte, tengo una relación cercana con otras personas)	5.75	1.56	–1.27	0.90	0.71	0.50
11. I show concern for others in my sport. (En mi deporte, muestro interés por los demás)	5.19	1.72	–0.78	–0.19	0.68	0.54
14. There are people in my sport who care about me. (En mi grupo de entrenamiento, hay personas que se preocupan por mí)	5.19	1.83	–0.79	–0.39	0.82	0.33
17. In my sport, there are people who I can trust. (En mi grupo de entrenamiento, hay gente en la que puedo confiar)	5.89	1.53	–1.46	1.45	0.81	0.34
20. I have close relationships with people in my sport. (Siento que me llevo bien con mis compañeros de entrenamiento)	5.52	1.79	–1.06	0.13	0.69	0.52

*The saturations greater than 0.60 are significant at *p* < 0.01; ^∗^*p* < 0.05; λ = weighting factor; δ = error term.*

**TABLE 3:** Phi correlation matrix between the five latent factors of the Basic Needs Satisfaction in Sport Scale (BNSSS).

	**1**	**2**	**3**	**4**	**5**
1. Competence satisfaction		0.55^∗∗^	0.37^∗∗^	0.70^∗∗^	0.64^∗∗^
2. Relatedness satisfaction	0.06		0.31^∗∗^	0.70^∗∗^	0.64^∗∗^
3. Perceived choice	0.13	0.09		0.17	0.43^∗∗^
4. Volition	0.49	0.49	0.02		0.89^∗∗^
5. IPLOC	0.40	0.40	0.18	0.79	

*^∗∗^*p* < 0.01; the scores in the left bottom part of the table are squared correlations.*

According to BPN, there was a second model of three-factors (Model 2) tested: competence satisfaction, relatedness satisfaction, and autonomy satisfaction, which is a latent construct conformed by the three observed qualities: volition, perceived choice, and IPLOC. The results showed a poor fit (Table 1). Moreover, the saturation of item 8 was low (λ = 0.07) and not significant. Therefore, an alternative model (Model 2A) was tested, and the item mentioned before was eliminated. The results showed an acceptable adjustment of the model to the data (Table 1), and when comparing the differences of the incremental adjustment indexes between Model 2 and Model 2A, a better fit for the alternative model was observed. Furthermore, all the items saturated significantly (*p* < 0.01) with factor loadings that ranged between 0.76 and 0.90 for competence satisfaction; between 0.68 and 0.82 for the relatedness satisfaction; and between 0.39 and 0.86 for autonomy satisfaction. The correlation between the latent factors was positive and significant between competence satisfaction, and autonomy satisfaction (φ = 0.67, *p* < 0.01); between autonomy satisfaction and relatedness satisfaction (φ = 0.66, *p* < 0.01); and between satisfying relatedness and competence satisfaction (φ = 0.55, *p* < 0.01).

### Internal Consistency and Convergent Validity

In this section, the emerging factors of the M1 and M2A models were analyzed considering the previous results. McDonald’s omega values were acceptable for all the factors, except for volition. In addition, the AVE values for all factors were adequate, except for the volition (see Table 4).

**TABLE 4:** Mean, standard deviation, composite reliability, and average variance extracted from the factors of the Basic Needs Satisfaction Scale in Sports.

	**Range**	***M***	***SD***	**ω**	**AVE**
Competence satisfaction	1–7	5.63	1.23	0.92	0.71
Relatedness satisfaction	1–7	5.50	1.26	0.86	0.55
Perceived choice	1–7	4.62	1.57	0.86	0.61
Volition	1–7	4.80	1.07	0.50	0.29
IPLOC	1–7	5.85	1.28	0.88	0.71
Autonomy satisfaction^1^	1–7	4.08	1.22	0.93	0.63

*^1^Composed by the observed qualities of volition, perceived choice, and IPLOC.*

### Nomological Validity

Based on the results of factorial and convergent validities, the five-factor structure of the BNSSS, plus the autonomy satisfaction factor (composed by the three observed qualities of autonomy), were related to theoretically associated constructs. Results showed that these factors were positively related to the enjoyment of participation in sports, and to the subjective vitality of the athletes (Table 5).

**TABLE 5:** Pearson’s Matrix correlations between the scores of the factors of the Basic Needs Satisfaction in Sport Scale (BNSSS) with related constructs.

	**ω**	**Range**	***M***	***SD***	**BNSSS**					
					
					**Competence**	**Relatedness**	**Choice**	**Volition**	**IPLOC**	**Autonomy^1^**
Enjoyment	0.79	1–5	4.08	0.64	0.38^∗∗^	0.37^∗∗^	0.19^∗∗^	0.47^∗∗^	0.52^∗∗^	0.44^∗∗^
Vitality	0.84	1–7	5.66	1.17	0.50^∗∗^	0.41^∗∗^	0.28^∗∗^	0.37^∗∗^	0.41^∗∗^	0.40^∗∗^

*^1^Composed by the three observed qualities of autonomy; *^∗∗^p* < 0.01.*

### Factorial Invariance

Regarding the results of factorial, convergent and nomological validities, the three-factor model’s (Model 2A) factorial invariance according to gender was proved. Initially, the factorial structure was acceptable for each group (men and women). As shown in Table 6, the results provided good data adjustment in both groups (M0a and M0b). Regarding the multisample models, the base model (M1), in which no equality restrictions were imposed. The results showed an acceptable adjustment; thus, the same three-factor model was able to adjust the data from each group.

**TABLE 6:** Adjusted goodness indexes of invariance models through gender.

**Model**	**Model description**	**χ^2^**	***df***	**RMSEA**	**NNFI**	**CFI**	**PGFI**	**ΔRMSEA**	**ΔNNFI**	**ΔCFI**
M0a	Base model Men	544.75^∗^	149	0.07	0.94	0.95	0.64			
M0b	Base model Women	426.18^∗^	149	0.08	0.90	0.90	0.63			
M1	Base model of structural invariance	804.18^∗^	298	0.07	0.94	0.95	0.64			
M2	Factorial weight invariance	872.21	319	0.08	0.92	0.93	0.61	<0.01	>0.01	>0.01
M2a	Partial factorial weight invariance	830.21^∗^	316	0.08	0.93	0.94	0.61	<0.01	<0.01	<0.01
M3	Partial factorial weight invariance and total intercepts	970.36^∗^	327	0.09	0.90	0.89	0.59	>0.01	>0.01	>0.01
M3a	Partial factorial weight invariance and partial intercepts	936.74^∗^	324	0.09	0.88	0.86	0.55	>0.01	>0.01	>0.01

*^∗^*p* < 0.01.*

Moreover, the M1 was used as a base to compare the following restricted models. The Model 2 (M2) tested the case that all factorial loads are invariable across gender. The fit indexes exhibited a reasonable adjustment. However, the differences between incremental indexes showed that the total invariance of the factor loadings could not be supported. A nested model on the latter M2A was tested where the saturation of factorial items with high indexes of modification were freed.

In the end, the Model 3 (M3) tested the partial invariance of the factor loadings and the total of intercepts. Practical adjustment indexes showed a reasonable adjustment. However, the incremental adjustment indexes did not confirm the hypothesis. Based on the modification indexes and on successive steps, the intercepts of items which showed higher modification indexes in the intercept parameter were freed, without achieving a model with satisfactory adjustment indexes (M3a), dismissing the partial invariance of the intercepts.

## Discussion

This paper aimed to adapt the BNSSS (Ng et al., 2011) to Spanish spoken in Mexico, and to analyze its psychometric properties (factorial validity, factorial invariance, internal consistency, convergent validity, and nomological validity) with a sample of child–adolescent athletes.

First, a five-factor structure was confirmed (perception of competence, perception of relatedness, IPLOC, volition, and perceived choice; the last three-factors are theorized as observed qualities of autonomy satisfaction). Although results suggest eliminating one volition factor item, the statistical significance shows it could be maintained, and that its elimination would not bring any improvement to the model. The remaining items were appropriate for measuring the aforementioned constructs. The results agree with other studies that proved the five-factor structure (e.g., Ng et al., 2011; De Francisco et al., 2018). Nevertheless, of the original English version, carried out with a young adults’ sample (Ng et al., 2011), the results evidenced that three items of different factors saturated below the criterion, while in our study only one of the items, volition factor, had a low factor loading. The same item also showed a low factor loading in the study of De Francisco et al. (2018) with a sample of teens and adults.

In the five-factors structure, the volition and IPLOC factors correlate strongly, so it cannot be supported that the items that compose them discriminate between both factors, since it is widely accepted that discriminant validity can be established when the correlations between the factors are below 0.85 (Kline, 2005). This suggests that the two subscales measure similar constructs this means that there is only one construct. This result is similar to the original version in English (Ng et al., 2011), and its Spanish adaptation (De Francisco et al., 2018). Moreover, the reliability of the volition factor was inadequate, which leads to high measurement errors; while the AVE for this factor revealed that less than half of the indicator’s variance is explained by the model. Thus, the group of items proposed for this construct is imprecise to measure. This result is similar to the obtained by Ng et al. (2011) in its original English version, since the volition factor had the lowest reliability (Cronbach’s alpha = 0.61). In the present study, the other factors presented an adequate composite reliability, because it ranked above the criterion for the scales in psychological matters, and because the AVE was acceptable.

Second, considering the three BPN and other studies (e.g., Andrade et al., 2018), a model of three-factors was tested – relatedness satisfaction, competence satisfaction, and autonomy satisfaction (composed by the observed qualities of volition, perceived choice and IPLOC). The three-factor model adjusted acceptably after deleting an item. This item was also problematic in the adaptation to Spanish (De Francisco et al., 2018) with a lower value of *R*^2^.

The five-factor model and the three-factor model were adequate. However, the convergent validity suggests that three-factor structure is better, because the AVE values show that all indicators are valid, which means that more than half of the variance observed in the items was accounted for their hypothesized factors; which did not happen in the volition construct (five-factor model). Also, the results indicate that these constructs achieved discriminant validity since the AVE values were above the squared inter-correlations, which did not happen in the correlation between IPLOC and volition (five-factor model). Moreover, the composite reliability of the three constructs was adequate. Adding the observed quality of the latent construct of autonomy satisfaction, both, the internal consistency and the AVE tend to improve. Therefore, measuring the autonomy satisfaction through the combination of the qualities of volition, choice and IPLOC is better than measuring them separately.

In addition, the nomological validity shows that the satisfaction of BPN and the qualities of autonomy are related positively and significantly, with subjective vitality and with the enjoyment of sports participation, which agrees with other studies (Quested et al., 2013; González et al., 2015; Garza-Adame et al., 2017; Monteiro et al., 2018). Volition and IPLOC factors show a similar pattern of correlation with subjective vitality, and the enjoyment of sports participation, while the choice factor shows the same pattern, but with lower coefficients. This agrees with Reeve et al. (2003), who found that the scores derived from the IPLOC and volition subscales determined a similar relationship with the related constructs.

Although other research in sports has measured the IPLOC and perceived choice as different facets of autonomy (Reinboth and Duda, 2006), the results of factorial and convergent validities support the integration of IPLOC and volition aspects as valid indicators within the latent construct of autonomy in this work. Reeve et al. (2003) evidenced that these qualities may overlap in the educational context, and it happens in the sports one. The results of nomological validity supported that the quality of perceived choice behaves as an epiphenomenon within the autonomy construct (Deci and Ryan, 1985). This may happen because the experience of choice can be understood in cognitive and motivational concepts (Deci and Ryan, 1985). A cognitive concept occurs when a person decides or chooses to do something among some options and not by a real sense of choice. In the other hand, a motivational concept applies only when a person feels free to choose. This suggests that it is necessary check the items of perceived choice, so that they are designed in a way that increases volition and IPLOC, that is, items on free choice, rather than providing choices.

In general, the results from this work suggest that the division of autonomy in three qualities (IPLOC, volition, and perceived choice) is more artificial than real. They support that IPLOC and volition are central qualities in the autonomy, suggesting that people experience these qualities as an overlap, while perceived choice behaves as an epiphenomenon (Deci and Ryan, 1985) that operates in an independent way.

Finally, the results of the multisample CFA, carried out with the three-factor model without an item, the model partially accepts invariance (i.e., weak and strong factorial criterion). However strict criteria were not met. The factorial saturation and the intercept of items 12, 13, and 19, regarding the autonomy satisfaction factor, vary across gender. This means that they have a non-uniform differential operation, in other words they discriminate better for men than for women. The rest of the items show a uniform differential operation.

This study has theoretical and practical implications. From the theoretical point of view, several structural models have been tested for BNSSS, contributing to the validity of the three-factor model. The invariance across gender supports the use of the scale to measure differences in BPN satisfaction across men and women, and the significant comparison in the observed scores. However, more studies are needed to confirm the total factorial invariance of the scale; to justify the use of this instrument; to follow differential analysis across groups; and to allow an impartial separation of average scores across men and women (Moreno-Murcia et al., 2017).

This study was developed upon a sample of child and teenager athletes. These age groups are commonly used for validating instruments in physical education, but rarely in sports. It is necessary to focus in these age range of 10–12 years to understand the motivational processes in childhood-adolescence transition (Méndez-Giménez et al., 2016). Ryan and Deci (2002) pointed out how important to all ages and cultures the application of theoretical objectives should be. The transcultural adaptation of situations that cover those measures is important, since the translation and adaptation of the test is of great interest in psychometric research (Muñiz et al., 2013) that facilitates the comparative and intercultural research.

From the practical point of view, the validation of the BNSSS in Spanish spoken in Mexico represents a development in Mexico’s sport psychology, because we have adapted a single sport-specific measure of the three needs, which was not available in this context. One of the strengths is that an individual and team sports sample was used, this will provide an instrument for researchers and psychologists in México to evaluate the extent in which the BPN in sports are met, and how these relate to other variables, so that they can subsequently design interventions that favor athletes’ positive experiences and performance.

This work also had its limitations, such as the small sample size. In addition, only athletes of some sports participated, so it is not possible to generalize the results to all sports. The results should be taken with caution, since the sample included athletes of child and adolescent age, and that does not guarantee its extension to other age groups, since the results could be influenced by the understanding of the items. Therefore, it is suggested to replicate the study with samples that include more sports, and other age groups, because Messick (1995) argued that the construct validation is a continuous process, and that the evidence must be collected from a number of different samples to evaluate the psychometric properties of an instrument adequately.

## Conclusion

The results of this study, based on the scores from the BNSSS adapted at the Spanish spoken in Mexico, show good psychometric properties of this scale, and provides evidence that it is adequate to measure the satisfaction of the three BPN theorized by the SDT (autonomy, competence, and relatedness) after the elimination of an item.

## Data Availability Statement

The datasets generated for this study are available on request to the corresponding author.

## Ethics Statement

The studies involving human participants were reviewed and approved by the Local Committee for Research and Ethics in Health Research. Written informed consent to participate in this study was provided by the participants’ legal guardian/next of kin.

## Author Contributions

HP-E was responsible for all aspects of the work to identify that issues related to its accuracy or integrity are resolved problems, designed the work, wrote the manuscript, and reviewed the intellectual content. IL and SM made substantial contributions as they were responsible for the back translation, and together with AG critically reviewed the style of American English. VM-S and RM-S were the two experts in sports psychology, and helped to acquire the data. ED and AG contributed to the concept and design of the work, since they were the experts in the sport reviewing the adaptation of the instrument. VM-S interpreted the data. All authors have approved the final version of the manuscript to be published.

## Conflict of Interest

The authors declare that the research was conducted in the absence of any commercial or financial relationships that could be construed as a potential conflict of interest.
